# Supplementary material for: “It’s hard to keep a distance when you’re with someone you really care about”—A qualitative study of adolescents’ pandemic-related health literacy and how Covid-19 affects their lives
Source: PLoS One. 2022 Apr 1;17(4):e0266510. doi: 10.1371/journal.pone.0266510 (PMC8975157; doi:10.1371/journal.pone.0266510)
Supplement: S1 File — (DOCX) [file pone.0266510.s001.docx]

**Interview guide**

| **Topic** | **Questions** |
| --- | --- |
| *Hello* | Can you tell us a little bit about yourself and what you like to do? |
| *Information about protective measures* | Can you tell us about the current guidelines for your school and municipality?  Where do you find information about corona and protective measures? Is finding information hard or easy?  In media there are a lot of information on corona and prevention. Does this make you confused? What do you do if you get uncertain about information? How do you assess the information?  How do you experience your family, friends and people around you to deal with information, guidelines and rules? |
| *Preventive behavior* | What are your thoughts on hand washing? Has your hand washing behavior changed since the beginning of the pandemic? How?  What do you feel about following this rule (keeping one meter apart)? Does this have consequences as to how you behave? How?  At the beginning of the pandemic we were asked to meet few people and many chose a small group to hang out with. How did you relate to this and what were your experiences? Are you still seeing less friends than normal? How do you meet with friends now? What are your experiences now? |
| *Quality of life* | What is it about the current situation that affects you the most (leisure time activities, school, friends, physical activity, emotions)?  Is following the rules related to how you feel (your quality of life)? Can you elaborate? During the pandemic, do you think that people may feel lonely? Do you miss anything? What do you miss the most? Has this changed since the first lockdown? Do you have any thoughts on how the pandemic may affect your future? Is there anything positive coming from this situation? In what way? |
